# 3D Printing High-Consistency Enzymatic Nanocellulose Obtained from a Soda-Ethanol-O_2_ Pine Sawdust Pulp

**DOI:** 10.3390/bioengineering6030060

**Published:** 2019-07-16

**Authors:** Heli Kangas, Fernando E. Felissia, Daniel Filgueira, Nanci V. Ehman, María E. Vallejos, Camila M. Imlauer, Panu Lahtinen, María C. Area, Gary Chinga-Carrasco

**Affiliations:** 1 VTT Technical Research Centre of Finland Ltd., P.O. Box 1000, FI-02044 VTT, Finland; 2 Instituto de Materiales de Misiones (IMAM), Félix de Azara 1552, 3300 Posadas, Misiones, Argentina; 3 RISE PFI, Høgskoleringen 6b, 7491 Trondheim, Norway

**Keywords:** pine sawdust, soda ethanol pulping, nanocellulose, 3D printing, cytotoxicity

## Abstract

Soda-ethanol pulps, prepared from a forestry residue pine sawdust, were treated according to high-consistency enzymatic fibrillation technology to manufacture nanocellulose. The obtained nanocellulose was characterized and used as ink for three-dimensional (3D) printing of various structures. It was also tested for its moisture sorption capacity and cytotoxicity, as preliminary tests for evaluating its suitability for wound dressing and similar applications. During the high-consistency enzymatic treatment it was found that only the treatment of the O_2_-delignified pine pulp resulted in fibrillation into nano-scale. For 3D printing trials, the material needed to be fluidized further. By 3D printing, it was possible to fabricate various structures from the high-consistency enzymatic nanocellulose. However, the water sorption capacity of the structures was lower than previously seen with porous nanocellulose structures, indicating that further optimization of the material is needed. The material was found not to be cytotoxic, thus showing potential as material, e.g., for wound dressings and for printing tissue models.

## 1. Introduction

Balancing environment, biodiversity, and economic development can be performed through ecosystem services, climate change mitigation strategies, and long-term food security balance. In this context, the possibility of recycling and reusing a resource represents an issue of fundamental importance in the economy of various industrial sectors. The biorefinery of agro and industrial forestry waste implies its integral use and its valorization, being able to satisfy the needs of food, raw materials, and energy, respecting the principles of sustainability. More than 65% of the plantations in Argentina are in two provinces in the Nord-East (Misiones and Corrientes), of which approximately 60% is pine, since they have very favorable conditions for the growth of species such as *P. elliottii* and *P. taeda* (yields above 20 m^3^/year). Therefore, pine sawdust is the most important waste of primary wood processing. This waste is not used properly and its accumulation contributes to the pollution of the environment [1].

Fractionation is a sequence of processes, which allow for recovering the different chemical components of a raw material. Different methods have been investigated for pine sawdust fractionation, including hot water, dilute acid, steam explosion, acid organosolv, and traditional alkaline treatments like soda-anthraquinone and kraft [2,3,4,5,6,7,8]. This raw material has been proven to be very recalcitrant and acid treatments before the delignification stage produced alterations in the structure that made the fractionation even more difficult [9]. On the contrary, the use of organic solvents combined with bases seems to be promising. Ethanol reduces the surface tension of the pulping liquor favoring the alkali penetration into the material structure [10]. The organic solvent also alters lignin-carbohydrate bonds by hydrolyzing lignin which is then dissolved in the organophilic phase [11]. This processing produces a pulp enriched in cellulose and a less condensed lignin. In studies comparing acid processes for bioethanol production, the organosolv treatment presents higher energy consumption than the diluted acid treatment [12]. Nevertheless, in organosolv processes, a high-quality lignin can be recovered. On the contrary, if in addition to lignin a high-added-value product such as nanocellulose is produced, the process turns auspicious [13]. In addition to the above, the alkaline processes have a much stronger recovery system than the acid ones. Another strategy for further extraction of lignin from the lignocellulosic material after pulping is oxygen delignification, using oxygen and alkali in a pressurized system. Oxygen reactions are generated by radicals which react with lignin removing it in a fraction corresponding to 25% to 65% of the initial kappa number of the pulp [14]. Kappa refers to the lignin content in a pulp, the higher the kappa number the higher the lignin content.

Nanocelluloses are promising bio-based materials for numerous applications, either as replacement of traditional oil-based materials in existing products or in generating completely new materials and products. They can be roughly divided into three categories based on their production methods and properties: Cellulose nanofibrils (CNF), Cellulose nanocrystals (CNC), and bacterial cellulose (BC). Of these, CNF are manufactured by mechanical treatments, often combined with chemical or enzymatic pre-treatment, and different manufacturing methods result in materials with variable properties. Research around different production methods of nanocelluloses has continued actively for over a decade and resulted in pilot, pre-commercial, and even commercial plants all over the world. Despite the rapid development, some challenges still remain with the traditional production technologies. Considering the mechanically manufactured nanocelluloses, the production costs are usually still high, and the resulting material is at low consistency, typically between 1%–3%. The high-water content generates problems, such as difficulties in dewatering and drying, problems in post-treatment, and restricted applicability for certain applications, such as composites or paints and coatings. In addition, long-distance transportation is not feasible leading to limited availability of the material.

To overcome the problems related to high energy consumption of nanocellulose manufacturing and low solids content of the resulting material, a high-consistency enzymatic fibrillation (HefCel) technology has been developed [15]. The benefits of the HefCel nanocellulose are its high-consistency after processing (10%–25%) and lower energy consumption compared to other manufacturing methods [16]. However, similar to other nanocelluloses, HefCel nanocellulose is a potential raw material for many different applications, such as strength additive in the middle ply of board [16] or as a barrier film in packaging materials [17].

During the last years, nanocellulose has been in the focus as a component in bioinks for three-dimensional (3D) printing [18]. The material has shear thinning behavior and consolidates rapidly after deposition on a substrate. These characteristics make it possible to deposit nanocellulose layer-by-layer in order to construct geometrically complex 3D objects. Rees et al. demonstrated the potential of nanocelluloses to be 3D-printed for constructing porous structures with potential as wound dressing materials [19]. Although nanocellulose with appropriate rheological properties are 3D printable, nanocellulose constructs require a post-treatment to be mechanically stable. Nanocellulose inks are usually combined with additional polymers such as alginate and cross-linked with Ca^2+^, thus yielding a mechanically stable 3D construct [20] with potential as scaffolds for tissue engineering. The composition of inks for 3D printing can be optimized with additional components to tailor the printability and shape fidelity, and to keep the stability at room temperature [21]. Most of the work on nanocellulose for 3D printing has been based on wood nanocellulose from market chemical pulp. However, recently it has been demonstrated that agro-industrial residues such as bagasse also have potential as resources for nanocellulose production and 3D printing [22].

The purpose of this work was to demonstrate and prove the suitability of a soda-ethanol pulping to yield fibres with adequate composition for production of nanocellulose by high-consistency enzymatic fibrillation, which in turn has appropriate properties for 3D printing. In addition, the cytotoxicity of the produced nanocellulose was tested to evaluate its potential suitability for wound dressing and similar applications.

## 2. Materials and Methods

### 2.1. Forestry Residues

*Pinus elliotti* and *Pinus taeda* sawdust mix was provided by a local sawmill (Forestal Eldorado and Forestal AM, Misiones). The sawdust was air-dried, screened, and maintained in closed plastic bags. The fraction passing 5 mm^2^-screen was used. 

Before each experiment, sawdust samples were impregnated with water overnight to eliminate bark particles by flotation.

Acid-insoluble lignin (Klason lignin) and pulp structural carbohydrates were measured according to the “Determination of Structural Carbohydrates and Lignin in Biomass” NREL/TP-510-42618. Hydrolysate samples from the aforementioned technique were neutralized with Ba(OH)_2_ following the methodology proposed by Kaar et al., HPLC with a SHODEX SP810 column was used to determine the carbohydrates content (glucans, xylans, mannans, galactans, and arabinans) [23]. The operational conditions used were water as eluent, 0.6 mL/min, 85 °C, and a refractive index detector. The final chemical composition of the pulp was completed by using an Aminex-HPX87H column (BIO-RAD), operated under the following conditions: 4 mM of H_2_SO_4_ as eluent, 0.6 mL/min, 35 °C, and a diode array detector.

### 2.2. Soda Ethanol Pulping

Soda-ethanol pulping was performed in a 7 L pressurized reactor (M/K Systems, Inc., Peabody, MA, USA), with direct heating and liquor circulation. About 500 g of dry sawdust was cooked. The pulping conditions are shown in Table 1. After the pulping stage, the spent liquor was separated from the pulp by filtration. The pulp was subjected to a 5-cycle washing with water, screened by means of a Somerville device, and properly stored in plastic bags. 

Residual alkali was measured from the black liquor according to the SCAN-N 33:94 method (“Residual Alkali – Hydroxide Ion Content”), and alkali consumption (%) was calculated. The Kappa number was determined following the TAPPI T236 om-99 procedure and yield was determined.

### 2.3. Oxygen Stage

The soda-ethanol pulps were treated with oxygen in two stages. Oxygen stages were conducted in a multipurpose reactor, equipped with a high shear rotor which generates the required agitation conditions, an oxygen inlet valve, and a heating system. In both stages the pulp was treated at 100 °C for 60 min, an oxygen pressure of 600 KPa and a consistency of 10%. The alkaline load was 3% on dry pulp in the first stage and 2% on dry pulp in the second stage. 

### 2.4. Chemical Composition of the Pine Sawdust Pulps

The content of acetone extractives was analysed according to standard SCAN-CM 49:03 and the lignin content and carbohydrate composition according to SCAN-CM 71:09. The samples were freeze-dried prior to analysis. The metal (Si, Fe, Mg, Mn, Co, and Ca) contents of the pulps were determined after wet combustion by inductively coupled plasma optical emission spectrometry (ICP-OEP).

### 2.5. Enzymatic Fibrillation

Two different types of pine sawdust pulps, namely soda-ethanol pulp and oxygen delignified (O_2_) soda-ethanol pulp, were used as raw materials for producing nanocellulose according to the HefCel technology. Prior to the treatments, the pulp samples were washed into Na^+^ form according to the method described by Lahtinen et al. [24]. The enzymatic treatment with cellulase mixture was carried out at a consistency of 25% dry weight (% odw) for 9 h (soda ethanol) and 5.5 h (O_2_ delignified soda-ethanol) at 70 °C and pH 5 using a two-shaft sigma mixer (Jaygo Incorporated, Randolph, NJ, USA) running at 25 rpm. The enzyme dosage in both treatments was 8 mg/g and the pulp batch size 300 g (% odw). After the treatments the enzyme was inactivated by increasing the temperature in the mixer to 90 ºC for 30 min. The fibrillated material was diluted with deionised water, filtered, and washed thoroughly with deionised water. Finally, the fibrillated material was dewatered to a consistency of 18.6% (soda ethanol HefCel) and 21% (O_2_ soda ethanol HefCel) by filtration. Yield of the fibrillated cellulose material was 91% for the soda ethanol HefCel and 88% for the O_2_ delignified soda ethanol HefCel. In order to prevent contamination prior to characterization, the HefCel materials were further autoclaved at 121 °C for 20 min and sealed. The materials were stored at +4 °C until used.

After initial testing by 3D printing, the O_2_ delignified soda-ethanol HefCel was further fluidized. Fluidization was done with a Microfluidics microfluidizer type M110-EH. Two passes were run through the chambers having a diameter of 400 µm and 100 µm at 1,800 bar operating pressure. The first pass was at 8% solids and the second pass was at 6% solids.

### 2.6. Characterisation of Nanocellulose

Preliminary characterization of the HefCel nanocellulose samples included pH, conductivity, dry matter content, optical microscopy, and rheological properties. A standard portable device Metler Toledo SG2 and Inlab 413SG electrode were used for the pH and Jenway 4510 for the conductivity measurement. Dry matter content measurement was based on oven drying.

#### 2.6.1. Apparent Viscosity and Yield Value

The shear viscosities of dilute HefCel nanocellulose samples were measured by a Brookfield rheometer model RVDV-III Ultra using vane-type spindles. The samples were diluted to 5% concentration with Milli-Q water and dispersed with an Ultra-Turrax disperser at 14,000 rpm for approximately 2 min. Viscosity measurements were performed in a 250 mL Pyrex beaker, and each sample was left to settle for a minimum of 30 min at room temperature after the dispersion. This allowed the samples to regain their initial viscosity. The temperature of the samples was adjusted to 20 ± 1 °C. The shear viscosity was measured at 300 measuring points at 0.5 rpm and at 180 measuring points at 10 rpm. The yield stress value was recorded at 0.5 rpm. The apparent viscosities and yield values were measured twice for each sample. Light mixing was performed between the measurements.

#### 2.6.2. Residual Fibre Analysis

Characterization of residual fibres from the HefCel nanocellulose samples was carried out with a Kajaani FibreLab analyser. Each sample was firstly soaked in 0.4 g/L consistency and dispersed using a high shear laboratory blender for 2–3 min. Then the samples were further mixed in 5 L of water at 40 mg/L consistency with an impeller for 10 min. Some 50 mL of dispersed sample or 2 mg in dry weight was pipetted into the analyser and fibre analysis was performed with two repeat measurements. The number of fibres recorded by the analyser was divided by the sample amount, which gives the value for the residual fibres in the sample.

#### 2.6.3. Optical Microscopy Imaging

The HefCel nanocellulose samples were dyed with 1% Congo red solution by mixing nanocellulose and dye in a ratio of 1:1 and further diluting the dyed mixture on the microscope slide (2:1). Optical microscopy was performed with an Olympus BX 61 microscope equipped with WH10X-H eyepieces, fluorite objectives, and a ColorView 12 camera.

#### 2.6.4. Laser Profilometry

Structures of 20 mm × 40 mm were printed directly on microscopy slides (one layer), using a Regemat3D printing unit. The structures were printed with a conical nozzle (size 0.58 mm) and a flow speed of 3.0 mm/s. The structures were allowed to dry for one day at room temperature (23 °C). The microscopy slides were sputtered with a layer of gold for laser profilometry analysis. Ten laser profilometry images (10 × 10 mm, 1.0 µm resolution) were acquired randomly. The laser profilometry images were assessed with the SurfCharJ plugin (v. 1q) for ImageJ (v. 1.50i). The 3D plots were created with the Interactive 3D surface plot plugin (v. 2.4). 

### 2.7. Three-Dimensional (3D) Printing

The nanocellulose gel (fluidized HefCel, 5 wt%) was used as inks for 3D printing. The 3D printing was performed with a Regemat3D bioprinter (version 1.0), equipped with the Regemat3D Designer (version 1.8, Regemat3D, Granada, Spain). Grids having a diameter of 20 mm, a height of 2 mm, and space between the tracks of 2 mm were printed. The inks were kept at room temperature (25 °C) for 24 h before printing. The flow speed was 3 mm/s, using a 0.58 mm conical nozzle. Fluidized HefCel was also combined with alginate (20%) before printing following the same set-up described earlier in this section. After printing, the grids were left in a solution of CaCl_2_ (50 mmol) for 24 h to cross-link the structure. The grids without and with alginate were freeze-dried with a Telstar LyoQuest-83 for 24 h.

The freeze-dried samples were immersed in MQ water, and the water holding capacity of the 3D-printed grids was estimated according to Equation (1):

Moisture sorption capacity (%) = (Wt − Wo)/Wo × 100(1)

where, Wt is the weight of the grid at given time points and W0 is the weight of the dry grid.

### 2.8. Cytotoxicity

The cytotoxicity of extracts from soda-ethanol HefCel, O_2_ delignified soda-ethanol HefCel, and fluidized O_2_ delignified soda-ethanol HefCel was evaluated according to ISO 10993-5:2009 annex C (3-(4,5-dimethylthiazol-2-yl)-2,5-diphenyltetrazolium bromide (MTT) cytotoxicity test) with extraction according to ISO 10993-12:2012. The HefCel samples were tested at two occasions: first, the unbleached and O_2_ delignified HefCel nanocellulose samples and later, the fluidized O_2_ delignified HefCel nanocellulose.

The MTT test is based on the measurement of the viability of the cells via metabolic activity. Yellow water-soluble MTT is metabolically reduced in viable cells to a blue-violet insoluble formazan. The number of viable cells correlates to the colour intensity determined by photometric measurements after dissolving the formazan in alcohol. Extracts from the test item, positive and negative controls, as well as blanks (extraction vehicle not containing the test item but subjected to conditions identical to those to which the test item was subjected to during extraction) were added to a subconfluent monolayer of L929 mouse fibroblast cells and incubated for 24 h at 37 ± 1 °C in 5% ± 1% CO_2_. After incubation the extracts were removed and MTT solution was added to the cells which were incubated for an additional 2 h at 37 ± 1 °C in 5% ± 1% CO_2_. Following incubation, the MTT solution was removed, 2-propanol was added, and the plates were shaken rapidly. Finally, the absorbance was measured at 570 nm (reference wavelength 650 nm) and the viability of cells was calculated. Thermanox Plastic Coverslips, Art no 174934 (Thermo Scientific NUNC) were used as the negative control and Latex rubber, Gammex 91-325 (AccuTech Ansell) as the positive control. 

## 3. Results and Discussion

The chemical composition of the raw material, pine sawdust, is shown in Table 2. The amount of total carbohydrates (cellulose and hemicelluloses) were 62.45%, the rest of this material was composed of acid-insoluble lignin (29.16%) and extractives (2.27%).

The yields and kappa numbers of the unbleached and O_2_ delignified pulps are shown in Table 3. The O_2_ delignification reduced the residual lignin to almost half the kappa number of the unbleached soda-ethanol pulp in Stage 1, and to a third in Stage 2.

The chemical compositions of the unbleached soda-ethanol and O_2_ delignified pulp (from Stage 2) are summarized in Table 4. The O_2_ delignification removed almost 70% of total lignin in the unbleached pulp, which slightly increased the composition of carbohydrates in the O_2_ delignified pulps. The results show that a significant amount of lignin was removed from the pulp during O_2_ delignification, while very little changes were seen in the carbohydrate content or composition. Some metals, such as Si, Fe, and Mn, were removed from the pulp during the delignification treatment, while for some the amount increased.

### 3.1. Nanocellulose Characterisation

**Figure 1:**
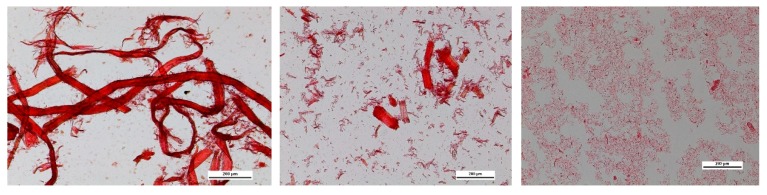
Optical microscopy images. (**left**) Unbleached HefCel, (**middle**) O_2_ delignified Hefcel, and (**right**) Fluidized O_2_ delignified HefCel. The bars are 200 µm.

Optical microscopy images of the HefCel nanocellulose samples provide information of their visual appearance, such as fibrillation degree and amount of unfibrillated fibres (Figure 1). Clearly, the degree of fibrillation was better in the case of the O_2_ delignified pulp and hardly any intact fibres could be detected. Unbleached material contained many more longer fibres after the enzymatic treatment. The bleached sample was cut into shorter fibre fragments (see Figure 1, middle) and thus, the amount of residual fibre particles was found to be higher according to the fibre analysis (Table 5). In the HefCel process, the accessibility of the enzyme to the cellulose fibre surface is a prerequisite, therefore, the lignin content or the number of adhesives or other impurities cannot be too high in the starting materials. This is probably the reason for the differences observed between the unbleached and O_2_ delignified HefCel samples (Table 4, Figure 1, left and middle).

Fluidization had a significant effect on the quality of the HefCel nanocellulose. When the O_2_ delignified HefCel sample was fluidized twice, the amount of residual fibre particles decreased to 5923 pieces (pcs)/mg from the original value of 49,703 pcs/mg. This change in the quality was also noticed in the microscopy image (Figure 1, right). The sample became more homogeneous and the low shear apparent viscosity increased from 487 mPa*s to 121,580 mPa*s when measured at the fixed 5% consistency.

**Figure 2:**
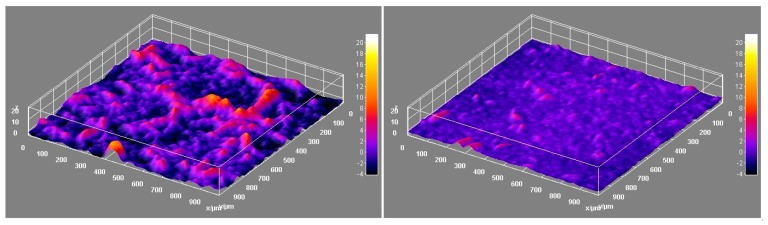
Images from laser profilometry. (**left**) O_2_ delignified Hefcel and (**right**) Fluidized O_2_ delignified Hefcel. The lateral size of the assessed areas was 1000 × 1000 µm. The z-direction calibration bar is between −4 and 20 µm.

**Figure 3:**
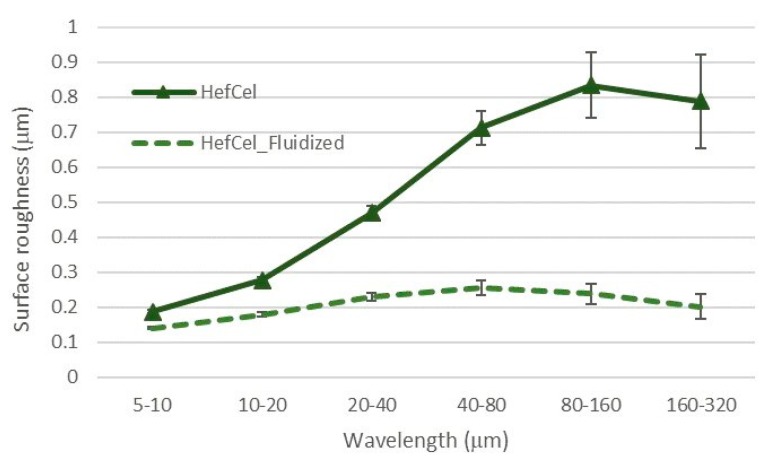
Laser profilometry. Surface roughness as a function of lateral wavelength. Note the large reduction of surface roughness after fluidization.

The laser profilometry analysis revealed the development of the HefCel nanocellulose samples before and after fluidization (Figure 2). The unfibrillated residual fibres observed in Figure 1 and detected by the residual fibre analysis (Table 5) caused a roughening of the surface structure of the films (Figure 2, left). The additional fluidization of the HefCel increased the fibrillation degree, thus reducing the amount of residual fibres. The surface roughness as a function of lateral wavelength clearly showed that the roughness decreased significantly after the fluidization (see wavelengths of 40 and 320 micrometers, Figure 3). This indicates a reduction of residual fibres and an increase of the nanofibril fraction [25].

### 3.2. 3D Printing

**Figure 4:**
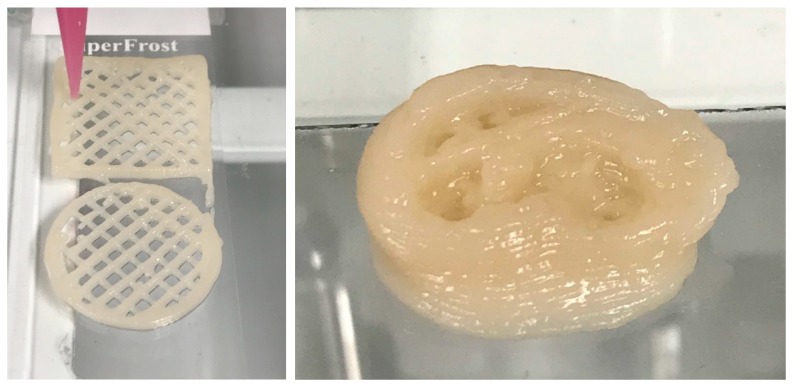
Three-dimensional (3D) printing of fluidized O_2_ delignified HefCel. (**left**) Circle (radius: 10 mm) and (**right**) squares (size: 20 mm × 20 mm) exemplifying the good print resolution and print fidelity. Right) Exemplification of a 3D-printed structure, i.e., a self-standing ear.

Various 3D structures were printed to verify the suitability of the fibrillated material to be deposited on a substrate layer-by-layer and form a 3D object (Figure 4). The unbleached and O_2_ delignified HefCel nanocelluloses were not printable, probably due to a relatively large fraction of residual fibres (Figure 1, Figure 2 and Figure 3, Table 5) that clogged the printing nozzle. However, after an additional homogenization with a microfluidizer the O_2_ delignified Hefcel sample performed well and the material could be extruded through a 0.4 mm nozzle (Figure 4). This behaviour was due to the additional fibrillation of residual fibres into micro- and nano-scale objects (Figure 3).

**Figure 5:**
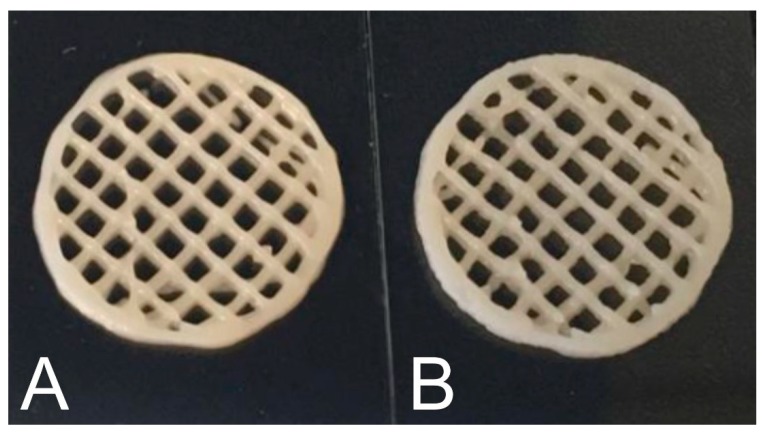
(**A**) Fluidized HefCel material. (**B**) Fluidized HefCel material and 20 wt% alginate. The radius of the circles is 10 mm.

**Figure 6:**
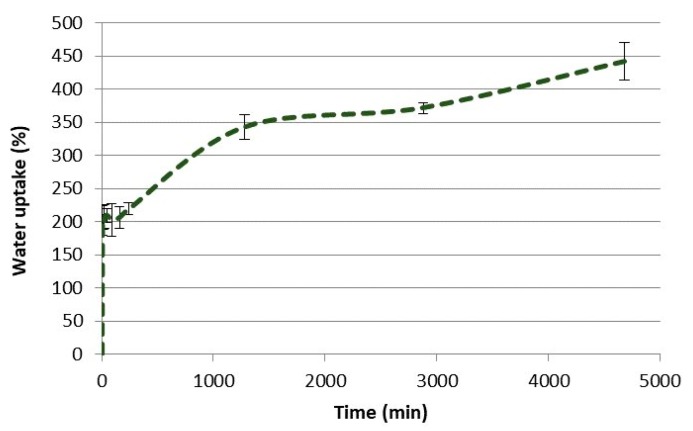
Water uptake of 3D-printed constructs containing HefCel, 20 wt% alginate and cross-linked with CaCl_2_. The water uptake was measured over 78 h.

Fluidized O_2_ delignified HefCel was also combined with alginate and cross-linked with calcium as a post-fixing step to solidify the structures (Figure 5). The 3D-printed grids were freeze-dried and immersed in water to assess the stability of the material and their water absorption capacity. The HefCel grid without alginate dissolved rapidly in water and it was not possible to quantify the water absorption capacity. The HefCel combined with alginate (20%) and cross-linked with Ca^2+^ performed well and the structure was stable even after several cycles of water immersion and lifting up to quantify the water absorption. The water absorption capacity reached a 450% weight increase after 78 h in water (Figure 6). This value is considerably lower than the previously reported values for porous nanocellulose structures, which reached levels of up to 10,000–16,000% water uptake [26,27] The relatively low water absorption capacity of the cross-linked HefCel sample was most probably due to two factors: (i) the relatively low fibrillation degree of HefCel and (ii) the cross-linking of the material which limits the water absorption. It is important to note that the water absorption capacity is one of the tests that are recommended for characterizing wound dressings [28].

### 3.3. Cytotoxicity

The tested samples (unbleached HefCel, O_2_ delignified HefCel, and fluidized O_2_ delignified HefCel nanocellulose) were found not to have cytotoxic potential according to the criteria given in the testing standard. The calculated cell viabilities are presented in Table 6. It is worth noting that the unbleached HefCel sample is just slightly above the limit to be considered not cytotoxic (70% cell viability). This indicates that this sample has a significantly lower cell viability compared to the O_2_ delignified and fluidized samples. The relatively lower cell viability caused by the unbleached HefCel sample may be caused by impurities and contamination that have not been removed by the pulping and nanocellulose production processes. The O_2_ delignified samples are subjected to more steps of processing and washing, which may have contributed to removing the potential impurities encountered in the raw material.

## 4. Conclusions

We provided an extensive characterization of pine sawdust pulp and produced high-consistency nanocellulose for 3D printing. O_2_ delignified soda-ethanol pulp was successfully fibrillated using high-consistency enzymatic fibrillation (HefCel) technology. However, a further homogenisation step by microfluidizer was needed to meet the requirements of 3D printing. After this step, various 3D structures of HefCel nanocellulose were successfully printed, with or without alginate. The structures containing alginate and cross-linked with CaCl_2_ were more stable against water. However, their water absorption capacity was lower compared to similar structures prepared from other types of nanocelluloses, possibly due to their limited fibrillation degree and use of a cross-linking agent. No cytotoxicity was observed for the HefCel materials studied, which is the first indication of the material suitability for wound dressing or similar applications.

## Figures and Tables

**Table 1:** Pulping conditions.

Parameter	Level
Maximum Temperature (T_máx._)	170 °C
Time at Maximum Temperature (t_máx._)	100 min
Time to Maximum Temperature (t_heating_)	60 min
Alkaline load (AL)	23.3% odw
Ethanol:Water Ratio (EtOH:H_2_O)	35:65% *v*/*v*
Liquor:Wood Ratio (L:W)	5.44:1

% odw: oven dried weight on mass of dry wood; *v*/*v*: percentage in volume.

**Table 2:** Chemical composition of pine sawdust.

Composition	% odw	StD
Ash	0.04	0.01
Total Extractives	2.27	
Extractives in Ethanol	1.54	0.03
Extractives in Water	0.73	0.01
Acid-Insoluble Lignin	29.16	0.10
Total Carbohydrates	62.45	
Glucans	40.30	0.38
Xylans	6.29	0.10
Galactans	2.18	0.08
Arabinans	0.77	0.02
Mannans	11.69	0.16
Acetyl Groups	1.22	0.02

% odw (% on mass of dry wood); StD (standard deviation).

**Table 3:** Yield and kappa number of soda-ethanol pulps.

Pulp	Stage *n*	Yield (%)	Kappa Number
**Unbleached Soda-ethanol Pulp**		43.6	29.9
**O_2_ delignified pulps**	Stage 1	97.4	13.8
	Stage 2	96.5	9.5

**Table 4:** Chemical composition of soda-ethanol pulps.

Chemical Composition	Unbleached Pulp	O_2_ Delignified Pulp (Stage 2)
Total Lignin (%)	4.1	1.2
Klason lignin (%)	3.7	0.7
Acid soluble lignin (%)	0.4	0.5
Acetone extract (%)	0.05	0.05
Total carbohydrates (%)	96.1	97.6
Arabinose (%)	0.4	0.4
Galactose (%)	0.5	0.3
Glucose (%)	82.9	84.1
Cellulose (%)	74.6	75.7
Xylose (%)	8.5	8.3
Xylan (%)	7.5	7.3
Mannose (%)	7.7	7.3
Mannan (%)	6.9	6.6
Si, mg/kg	109	60.1
Fe, mg/kg	102	87.8
Mg, mg/kg	438	515
Mn, mg/kg	47.2	35.7
Co, mg/kg	<0,5	1.5
Ca, mg/kg	1080	1180

**Table 5:** Characteristics of unbleached and bleached HefCel samples.

Sample	Yield Value (Pa)	StD	Apparent Viscosity, 10 rpm (mPa*s)	StD	Residual Fibres (pcs/mg)	StD
Unbleached HefCel	55	1	5853	337	31,310	34
O_2_ delignified HefCel	0.4	0.1	487	14	49,703	2
Fluidized O_2_ Delignified HefCel	314	13	121,580	1851	5923	115

**Table 6:** Calculated average cell viabilities and cytotoxicity grading according to ISO10993-5:2009.

Sample	Viability, % (Average)	STD	Cytotoxicity Grading
Unbleached HefCel	72.9	2.8	Not cytotoxic
O_2_ delignified HefCel	95.1	6.3	Not cytotoxic
Fluidized O_2_ delignified HefCel	96.9	1.4	Not cytotoxic
Positive control	1.1	0.1	Cytotoxic
Negative control	106.3	4.8	Not cytotoxic
